# Comparative microstructural study on the teeth of Mesozoic birds and non-avian dinosaurs

**DOI:** 10.1098/rsos.230147

**Published:** 2023-05-17

**Authors:** Yan Wang, Zhiheng Li, Chun-Chieh Wang, Alida M. Bailleul, Min Wang, Jingmai O'Connor, Jinhua Li, Xiaoting Zheng, Rui Pei, Fangfang Teng, Xiaoli Wang, Zhonghe Zhou

**Affiliations:** ^1^ Institute of Geology and Paleontology, Linyi University, Linyi, Shandong 276000; ^2^ Tianyu Natural History Museum of Shandong, Pingyi, Shandong 273300; ^3^ Key Laboratory of Vertebrate Evolution and Human Origins of Chinese Academy of Sciences, Institute of Vertebrate Paleontology and Paleoanthropology, Chinese Academy of Sciences, 142 Xi-zhi-men-wai Street, Beijing 100044; ^4^ National Synchrotron Radiation Research Center, Hsinchu 30076; ^5^ Department of Geosciences, National Taiwan University, Taipei City 10617; ^6^ Negaunee Integrative Research Center, Field Museum of Natural History, Chicago, IL 60605; ^7^ Key Laboratory of Earth and Planetary Physics, Institute of Geology and Geophysics, Innovation Academy for Earth Science, Chinese Academy of Sciences (IGGCAS), Beijing 100029; ^8^ Xinghai Paleontological Museum of Dalian, Dalian, Liaoning 116023

**Keywords:** bird, non-avian dinosaur, diet, tooth, dentin, enamel

## Abstract

Although it is commonly considered that, in birds, there is a trend towards reduced dentition, teeth persisted in birds for 90 Ma and numerous macroscopic morphologies are observed. However, the extent to which the microstructure of bird teeth differs from other lineages is poorly understood. To explore the microstructural differences of the teeth of birds in comparison with closely related non-avialan dinosaurs, the enamel and dentine-related features were evaluated in four Mesozoic paravian species from the Yanliao and Jehol biotas. Different patterns of dentinal tubular tissues with mineralized extensions of the odontoblast processes were revealed through the examination of histological sectioning under electron microscopy. Secondary modification of the tubular structures, forming reactive sclerotic dentin of *Longipteryx*, and the mineralization of peritubular dentin of *Sapeornis* were observed in the mantle dentin region. The new observed features combined with other dentinal-associated ultrastructure suggest that the developmental mechanisms controlling dentin formation are quite plastic, permitting the evolution of unique morphologies associated with specialized feeding behaviours in the toothed birds. Proportionally greater functional stress placed on the stem bird teeth may have induced reactive dentin mineralization, which was observed more often within tubules of these taxa. This suggests modifications to the dentin to counteract potential failure.

## 1. Introduction

The loss of teeth and associated dentinal tissues is a feature shared by crown birds (modern birds and their most recent common ancestor) [1]. As tooth reduction evolved independently multiple times within several lineages of Mesozoic avialans and even among non-avialan tetanuran theropods, the role of dentition in feeding was similarly considered to have become reduced [2,3]. During the evolutionary process leading to fully edentulous jaws and the acquisition of the rhamphotheca-covered bird beak, many Mesozoic stem avialan taxa lost teeth in parts of the jaw while retaining teeth in others [4]. For example, the tooth row in the premaxilla is shifted caudally with the cranial portion of the premaxillae edentulous, as seen slightly present in some fully toothed ornithuromorphs such as *Yanornis martiti* and *Yixianornis grabaui*, and maxillary teeth are lost and dentary teeth become restricted to the tip of the rostrum in the Longipterygidae [5,6]. In addition to the numerical reduction of the teeth themselves and restriction of the tooth row, changes are also observed within the internal microstructure and in the rate of tooth replacement during the early evolution and diversification of stem birds [7–9]. However, the key morphological and functional characteristics of Mesozoic avian dentition that distinguish them from closely related non-avian dinosaurs remain to be elucidated [9]. Although most Mesozoic enantiornithines and non-ornithothoracine avialans possessed jaws that at least partially retain teeth, the potential function these teeth played in feeding are largely enigmatic. Although initially bird teeth were thought to be simple conical structures without function, recent discoveries from the Jehol avifauna have revealed several taxa with unique enamel structures that strongly suggest the teeth in at least some taxa were functional and specialized for specific diets or feeding behaviours [10–13]. However, no enantiornithine preserves both the skull and *in situ* gut contents, thus preventing correlation between tooth morphology and diet. Even though they are present in other lineages, differences in the teeth between taxa with apparently similar diets undermines attempts to understand the role of tooth morphology in feeding, e.g. similar plant remains (seeds) are preserved in the crop of the ornithuromorph *Eogranivora edentulata* and the basal pygostylian *Sapeornis chaoyangensis*, but one is edentulous and the other has a specialized dentition [14]. Furthermore, the lack of suitable extant analogues further complicates attempts to understand the feeding ecology in Mesozoic toothed birds.

Earlier studies indicate that both tooth microstructure and macrostructure (e.g. the raw shape) vary between non-avian theropods and avialans, but how these differences relate to function remain ambiguous [15,16]. The Jehol enantiornithine *Longipteryx chaoyangensis* was originally inferred to be a piscivore based on its elongate rostrum [6]. This taxon also possesses a few large, distally recurved and crenulated teeth restricted to the distal portion of the rostrum. More recent interpretations suggest *Longipteryx* was insectivorous [17]. Direct evidence indicates that the Jehol basal pygostylian *Sapeornis* was herbivorous, interpreted as feeding on seeds with the aid of a gastric mill and large-sized premaxillary and maxillary teeth with specialized enamel grooves and tubercles and reduced dentary teeth that were possibly lost during ontogeny [3,13,18]. Also based on ingested remains, the small-sized paravian dinosaur from the Yanliao Biota, *Anchiornis huxleyi* is interpreted as an opportunistic carnivore that fed on lizards, possibly fish and other small vertebrates [19]. The volant, four-winged, Jehol dromaeosaurid *Microraptor* is also directly documented to have been an opportunistic carnivore, feeding on small mammals, birds, lizards and fishes, all recovered in different specimens [20,21]. Here, we investigate the ultramicrostructural characters of the teeth in the early avialans *Sapeornis* and *Longipteryx*, with detailed comparison with closely related paravian taxa, *Anchiornis* and *Microraptor*. The comparisons made here aim to provide insight into the different ecological adaptations at the origin of bird evolution [22]. Microstructural differences in morphology and the mechanisms controlling tooth growth and development across the dinosaur–bird transition can also be inferred from this sample of taxa. Tooth replacement, developmental mechanisms and reduction are all likely to be intimately related to physiological differences between non-avian dinosaurs and birds (i.e. the incubation time and embryonic growth rate, or digestive function) [23,24].

Both dentin and enamel microstructure of *Sapeornis* and *Longipteryx* were compared with those of the non-avialan dinosaurs *Microraptor* and *Anchiornis*. Scanning electronic microscopy (SEM), X-ray micro-computed tomography (µCT) and transmission electronic microscopy (TEM) are all applied in the tooth fragments or the sectioned slice in collecting the internal microscopic features (table 1). Enamel, dentin, cementum and alveolar bone were evaluated for all these taxa. The new data derived from both tooth and jaw materials confirm that the modification of the teeth in early birds involved both numeric reduction and microscopic modification [9]. These results highlight the need to not only investigate the tooth itself, but also the alveolar bone and cementum tissues and features affected by tooth replacement and development. Continued sampling of early birds and their closely related non-avialan dinosaurian relatives will be critical to track and better understand the modification of dental structures as they relate to dietary shifts and feeding ecology across the dinosaur–bird transition.

**Table 1.** Specimens and the samples examined and applied for each method; we sampled in total seven specimens referable to *Sapeornis*, *Longipteryx*, *Anchiornis* and *Mircoraptor*, including rostrum and isolated tooth materials.

species	specimen number	skull/ rostrum	isolated tooth	region of interest for electronic microscopic imaging	position of tooth selected
* Longipteryx chaoyangensis*	STM 8-92 IVPP 27103 DLXH 1079	CT-SEM	µCT	TEM, SEM [enamel, Dentin] SEM [cementum]	dentary/premaxilla
* Anchiornis huxleyi*	STM 0-69	CT-SEM	µCT	TEM, SEM [enamel, Dentin] SEM [cementum]	dentary
*Microraptor sp.*	STM 5-53 IVPP 28434	N/A	µCT	TEM, SEM [enamel, Dentin]	dentary
*Sapeornis chaoyangensis*	IVPP 19058	N/A	µCT	TEM, SEM [enamel, Dentin]	maxillae

Notes: institutional abbreviations: DLXH—Dalian Xinghai Natural History Museum; STM—Shandong Tianyu Natural History Museum; IVPP—Institute of Vertebrate Paleontology and Paleoanthropology.

## 2. Materials and methods

### 2.1. Specimens

The bird and dinosaur specimens were mainly chosen from the Institute of Vertebrate Paleontology and Paleoanthropology (IVPP) and Shandong Tianyu Natural History Museum (STM) for the selection of individual teeth. The rostrum was overall evaluated through CT-scanning to reveal the attachment and articulation of teeth within the jaw. Other sampled individual teeth of *Sapeornis*, *Longipteryx*, *Anchiornis* and *Microraptor* (table 1) were used for high-resolution µCT and electron microscopy imaging. Both SEM and TEM were applied to evaluate the internal dentin and enamel microstructures, with each suitable method applied for the isolated tooth, jaw materials and the whole skulls; the different methods applied are indicated in table 1. In this study, basal avialans *S. chaoyangensis* and *L. chaoyangensis* were selected because of previous proposed differences in their diets and feeding adaptations; the teeth were systematically evaluated through a combination of analytical methods including CT, SEM and TEM. The position of each tooth for sectioning was selected based on the availability of the teeth in the specimens and evaluation of relative destruction to the specimen due to sampling; selected tooth fragments included both dentary and maxillary teeth (provided in table 1).

### 2.2. Computed tomography scanning

CT scanning was applied for the overall examination of jaw material (with tooth) and performed on the Phoenix V|tome|x M300 instrument (GE, USA) which is a versatile X-ray microfocus CT system for three-dimensional metrology and analysis with up to 300 kV and 500 W. The rostrum with dentition *in situ* was scanned for the implantation, as well as the isolated teeth of *Sapeornis*, *Anchiornis*, *Longipteryx* and *Microraptor* is scanned (see electronic supplementary material, figures S1–S4). In addition, computed laminography was also applied on the whole jaw material with teeth and the scanning resolution range from 3 µm to approximately 70 µm based on the size of the samples (see electronic supplementary material, figure S3).

### 2.3. Scanning electronic microscope and transmission electronic microscopy experiments

For microstructural analyses with SEM and TEM methods, the tooth fragments of *Sapeornis*, *Longipteryx*, *Microraptor* and *Anchiornis* were sampled manually with a razor. One tooth was taken from a referred specimen of *Sapeornis* (IVPP V19058) and two (premaxilla and dentary) teeth fragments were taken from new specimens that are both referable to *Longipteryx* (IVPP V21703 and STM 8-92). The lower part of the isolated *Longipteryx* tooth was sectioned with targeted material including enamel and bulk dentin (electronic supplementary material, figure S3). Two dentary tooth fragments were taken from *Anchiornis* (STM 0-69) and two isolated dentary tooth fragments from the referred *Microraptor* specimen were sampled (STM 5-53 and IVPP 28434). All these sampled teeth fragments were embedded in epoxy resin using SPI-PON 812 Embedding Kit (MNA, EPOK, DDSA, DMP-30) [25]. After a stepwise polymerization at 37°C for 12 h, 45°C for 12 h, and 60°C for 48 h, the embedded samples were ground with a Leica EM TXP device and polished with a Lecia TIC 3X instrument in a step-wise manner for the smooth surface structural examination. The surface of the cross-section of some tooth samples were further etched with 0.1 mol l^−1^ phosphoric acid for approximately 0.5–2 min to visualize the crystallite structures, and then trimmed and cut with the Lecia (EM UC 7) machine equipped with a diamond knife (ultra 45°, 3 mm) for SEM and TEM observations. The thickness of TEM slices within the carbon-coated copper grid is approximately 70 nm, and the direction for cutting was mainly designed to be perpendicular to the long axis of tooth. SEM experiments on the cross-section of the tooth samples were conducted on a Quanta 450 FEI and a Haitech 3700 scanning electron microscope. TEM experiments were conducted on Talos F200 X transmission electron microscope (FEI) at the IVPP. This Talos F200X G2 is a 200 kV field emission gun (FEG) analytical scanning transmission electron microscope (S/TEM) equipped with highly sensitive four-detector Super-X EDS for quantitative elemental analysis and mapping (energy-dispersive X-ray EDX spectrometer), which allows for fast high-resolution imaging and X-ray microanalysis in both TEM and STEM modes.

## 3. Results

### 3.1. Enamel structure

**Figure 1.**
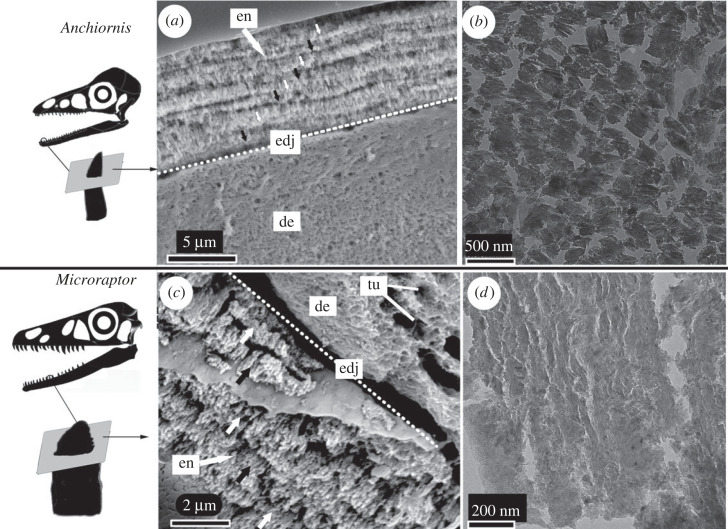
SEM and TEM images of the horizontal (labiolingual) cross-section of tooth in *Anchiornis huxleyi* (*a,b*) and *Microraptor gui* (*c,d*). The parallel organized crystallite enamel in *Anchiornis* is indicated by the alternating white and black arrows in (*a*); this structure is not clearly visible in *Microraptor* tooth (*c*). The arrangement of the enamel crystallite, visible under higher magnification through TEM (*b*,*d*). Abbreviations: de, dentin; en, enamel; edj, enamel dentin junction; tu, tubule. The selected teeth for sectioning are indicated in the schematic skull diagram on the left.

Enamel crystallites in *Anchiornis* are arranged in a parallel pattern visible through SEM (figure 1*a*), similar to those observed in other troodontids (e.g. *Byronosaurus*) and therizinosaurian dinosaurs [26]. The enamel measures approximately 6 µm in thickness with seven parallel incremental lines are recognizable in the horizontal cross-section (figure 1*a*: white and black arrows) [9]. Each incremental layer of the enamel is approximately 1 µm thick, similar to that of other small-sized theropods with a clear parallel crystallite pattern [15,27]. The enamel of the *Microraptor* tooth resembles that of other previously studied dromaeosaurids, e.g. *Velociraptor mongoliensis* [15], but differs from the *Anchiornis* tooth in that the crystallites are less organized although they retain an overall parallel arrangement (figure 1*c*). With high-resolution TEM, mineral crystallites were revealed by breakages of these enamel microunits due to the physical cut, which also reflect the overall paralleled pattern of the crystallite structure (figure 1*b,d*).

**Figure 2.**
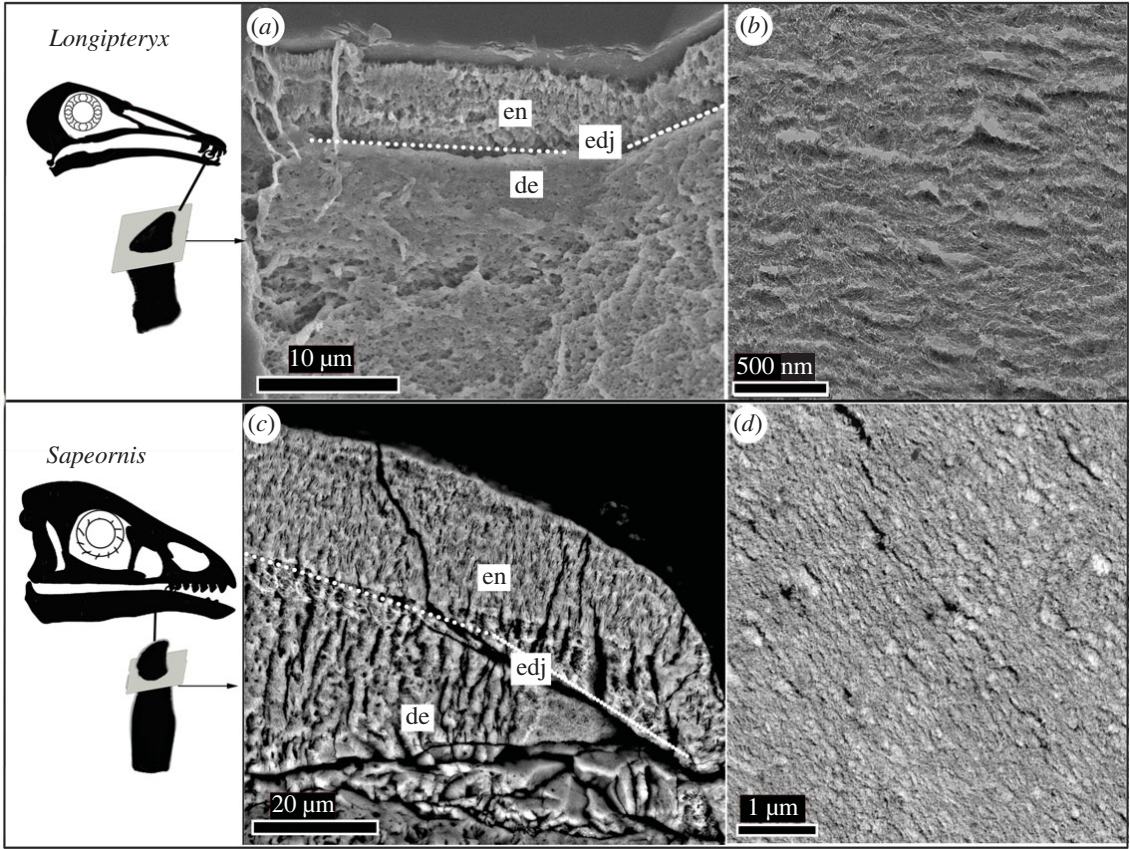
SEM and TEM images of the horizontal tooth cross-sections belonging to *Longipteryx chaoyangensis* (*a,b*) and *Sapeornis chaoyangensis* (*c,d*). The arrangement of the enamel crystallite was imaged through SEM (*a,c*) and TEM (*b,d*). Anatomical abbreviations are the same as figure 1. The premaxillary and maxillary teeth selected for the histological section are indicated in the schematic skull diagram on the left.

In comparison, the enamel crystallites in *Sapeornis* and *Longipteryx* are both vaguely arranged into columnar structures, but are mostly structureless (figure 2*b*,*d*). High-magnification TEM further indicates the lack of clear structure for the enamel crystallite in both *Longipteryx* and *Sapeornis* (figure 2). The orientation and size of the hydroxyapatite mineral (HAP) crystallite does not significantly differ between the two avian species.

### 3.2. Dentin structure

**Figure 3.**
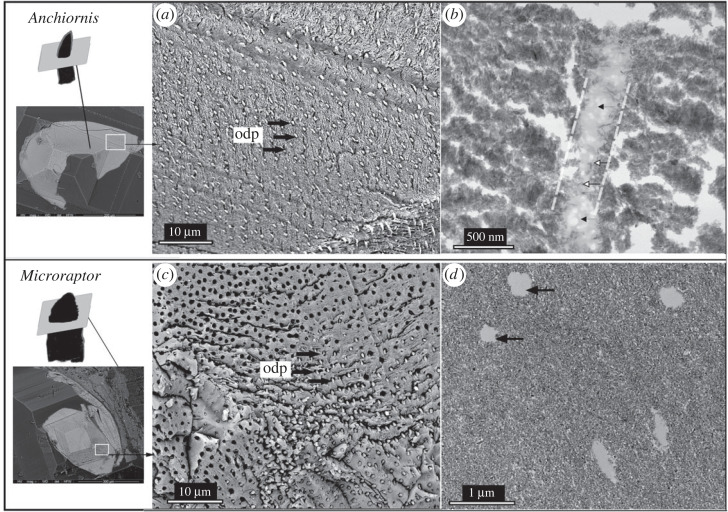
The dentin morphology of *Anchiornis* (*a,b*) and *Microraptor* (*c,d*). The black arrows indicate the odontoblast processes (labelled as ‘odp’) exposed in cross-section; some of the tubules are empty in the tooth section of *Microraptor*. In *Anchiornis*, the odontoblast processes are also observable through STEM image as amorphous tissues with weakly mineralized matrix vesicles (white arrow) and mineralized organic tissues (black arrow) in (*b*). The white dashed lines in (*b*) define the wall of one dentinal tubule; the black arrows in (*d*) indicate the empty tubules.

The average diameter of the dentinal tubules in *Anchiornis*, measured not far below the enamel–dentin junction (EDJ), is approximately 0.6–0.8 µm. This is smaller than that of all other troodontids and theropods examined previously, e.g. *Troodon formosus*, *Sinosaurus sp*. and *Tyrannosaurus sp*. [28]. A well-mineralized tubular-shaped material (mineralized cast of the odontoblast) was observed in the dentin of both *Anchiornis* and *Microraptor* under both SEM and TEM (figure 3*a*,*c*). The structure of the dentinal tubules is interpreted as the distal extension of odontoblast process [29].

**Figure 4.**
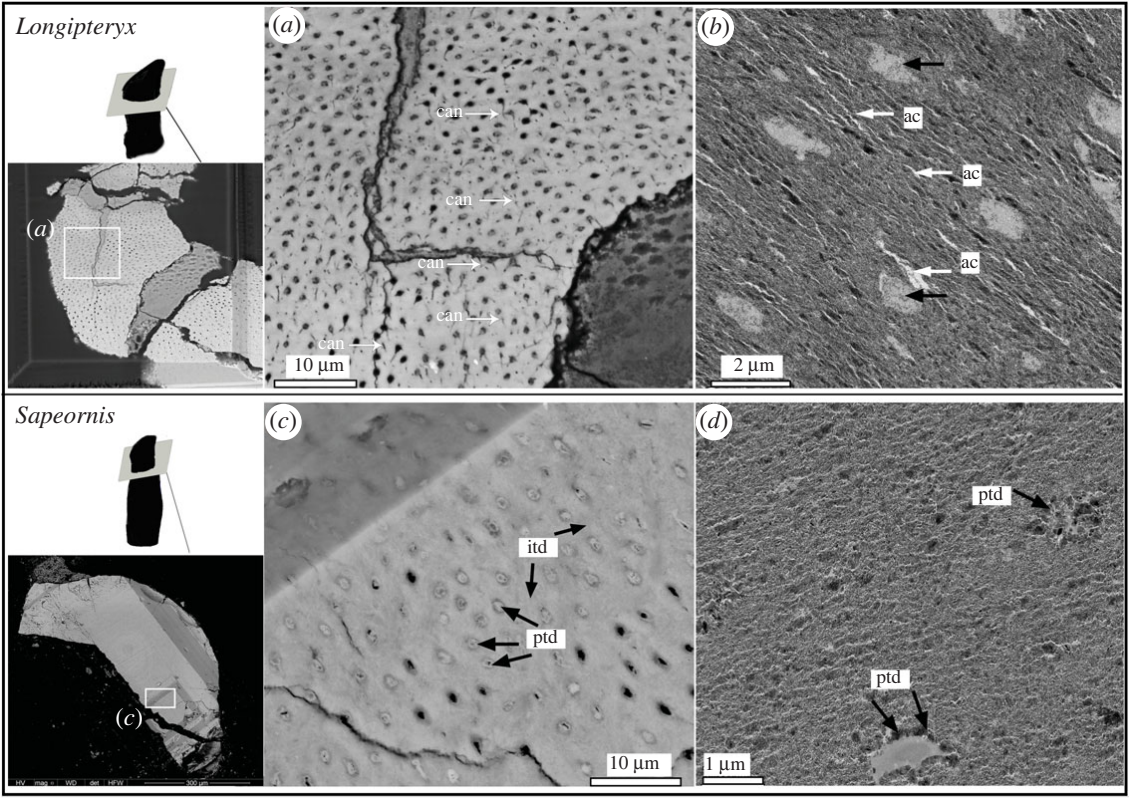
Microscopic features of dentin and dentinal tubules observed from cross-sections of the teeth in *Longipteryx* (*a,b*) and *Sapeornis* (*c,d*). SEM (*a*,*c*) and TEM (*b,d*) images of the dentinal tubules. Abbreviations: ac, artificial cracks formed during the cutting, which usually occurred close to the dentinal tubules (black arrows); can, canaliculi; itd, intertubular dentin; ptd, peritubular dentin.

In the stem bird samples, the lateral branches of the dentinal tubules (or ‘canaliculi’) were better developed in *Longipteryx* compared with the other taxa studied here (figure 4*a*: white arrows). The tubule shape within the *Longipteryx* tooth is distinct in having an elliptical shape, which may be caused by the slightly oblique direction of sectioning; the long axis of the elliptical tubules is slightly larger in *Longipteryx* (figure 4*b*) than that in *Anchiornis*, measuring over 1.0 µm compared with 0.6 µm in *Anchiornis*. The circular-shaped dentinal tubules in *Sapeornis* measure approximately 0.5–1.0 µm. The well-mineralized tubular wall within the dentinal tubules can be clearly observed in *Sapeornis* (figure 4*c*: ptd versus itd). The dense peritubular dentin (see ‘ptd’ in figure 4*c*) in *Sapeornis* was also confirmed through TEM, visible as a hyper-mineralized peritubular dentin that is denser than the intertubular dentin (figure 4*c*: ptd versus itd). The observed ultramicroscopic changes in dentin mineralization between taxa may be related to the adaptative differences in tooth hardness and elastic properties [30].

**Figure 5.**
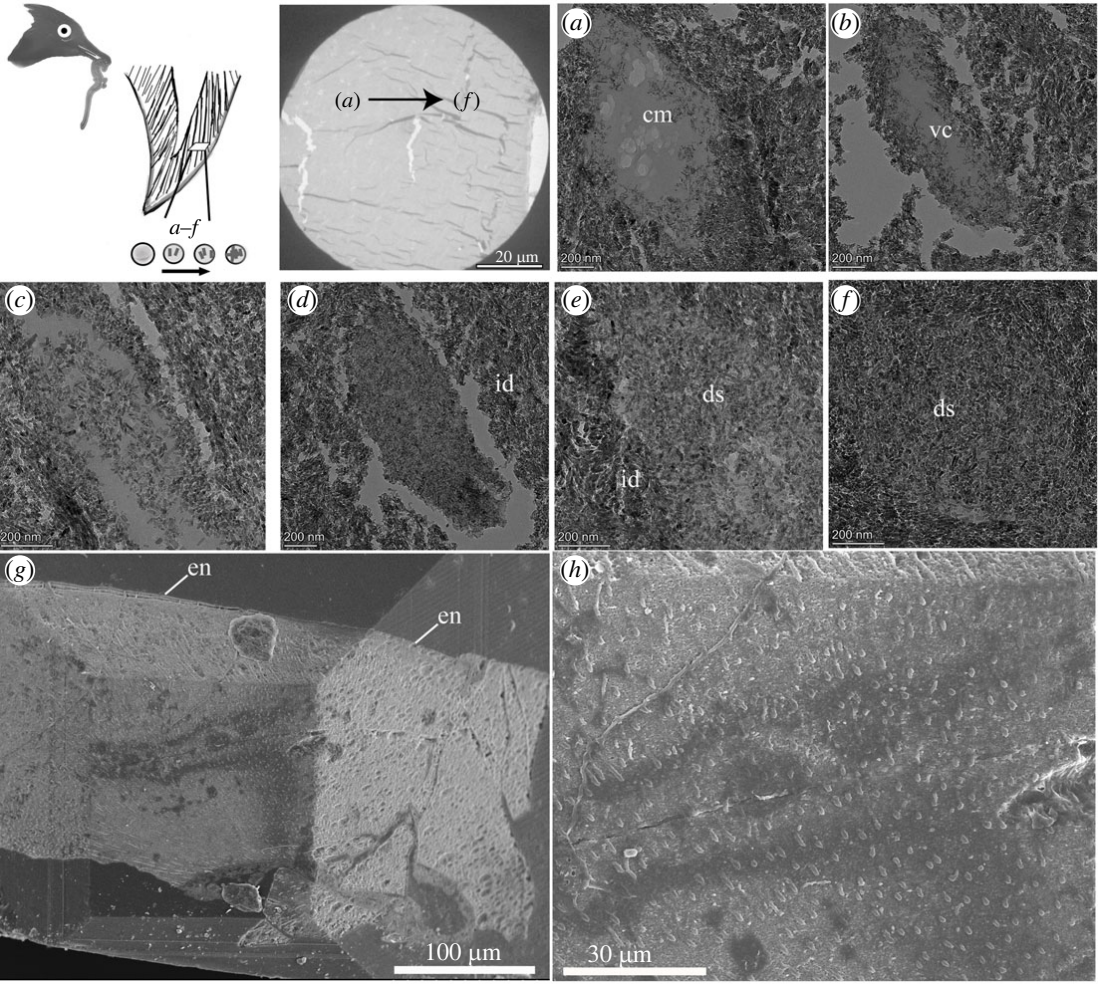
Ultramicroscopic structure of dentin tubular morphology of *Longipteryx* with a focus on the dentin region with ossified mineral infilling, the imaged area is indicated in the tooth diagram on the left as shown in the location close to the mantle dentin, not far beneath the enamel–dentin junction. The black arrow of the full-view image points in the direction of the exterior dentin with the serial layout of the high-magnification images arranged in (*a*)–(*f*), moving toward the enamel, with subset (*f*) being closest to EDJ. Nano-sized mineral crystals are observed in the tubules that were actively secreted from the organic matrix and fully occluded within some dentinal tubules. Abbreviations: cm, cytoplastic material; en, enamel; ds, dentinal sclerosis; id, interdentinal region; vc, vacuole. Potential predating behaviour was proposed for *Longipteryx* as shown in the art reconstruction.

TEM images reveal possible mineral infilling (during the life of the animal) of the dentinal tubules in *Longipteryx* (figure 5). The microscopic features of the dentinal tubules are interpreted as a large intracytoplasmic vacuole, and EDX mapping (electronic supplementary material, figure S1) supports the organic-rich area of these potential cellular structures preserved with HAP minerals secreted into the peri-odontoblastic space originally occupied by the organic cellular tissues (figure 5*a–f*). This secretion within the tubular tissues in the vacuole is similar to the distal extension of the odontoblast processes formed at the end [31]. The imaged section here captures the reactive (or tertiary) dentinogenesis process and similar tubular morphology was observed in the late stages of tooth formation, in which miniscule crystals form within the dentinal tubules in crown dentin [32]. The extensive mineralization and full obliteration of dentinal tubules are observed in *Longipteryx*, which is distinct from the homogeneous cast of tubular extension within the surrounding empty spaces observed in *Microraptor* and *Anchiornis* (figure 3). This may suggest plastic and secondary development of dentin during the later stages of tooth formation in *Longipteryx*, reflecting a response of dentinal tissue to extra stress that accumulates at the mantle dentin [9].

### 3.3. Cementum

**Figure 6.**
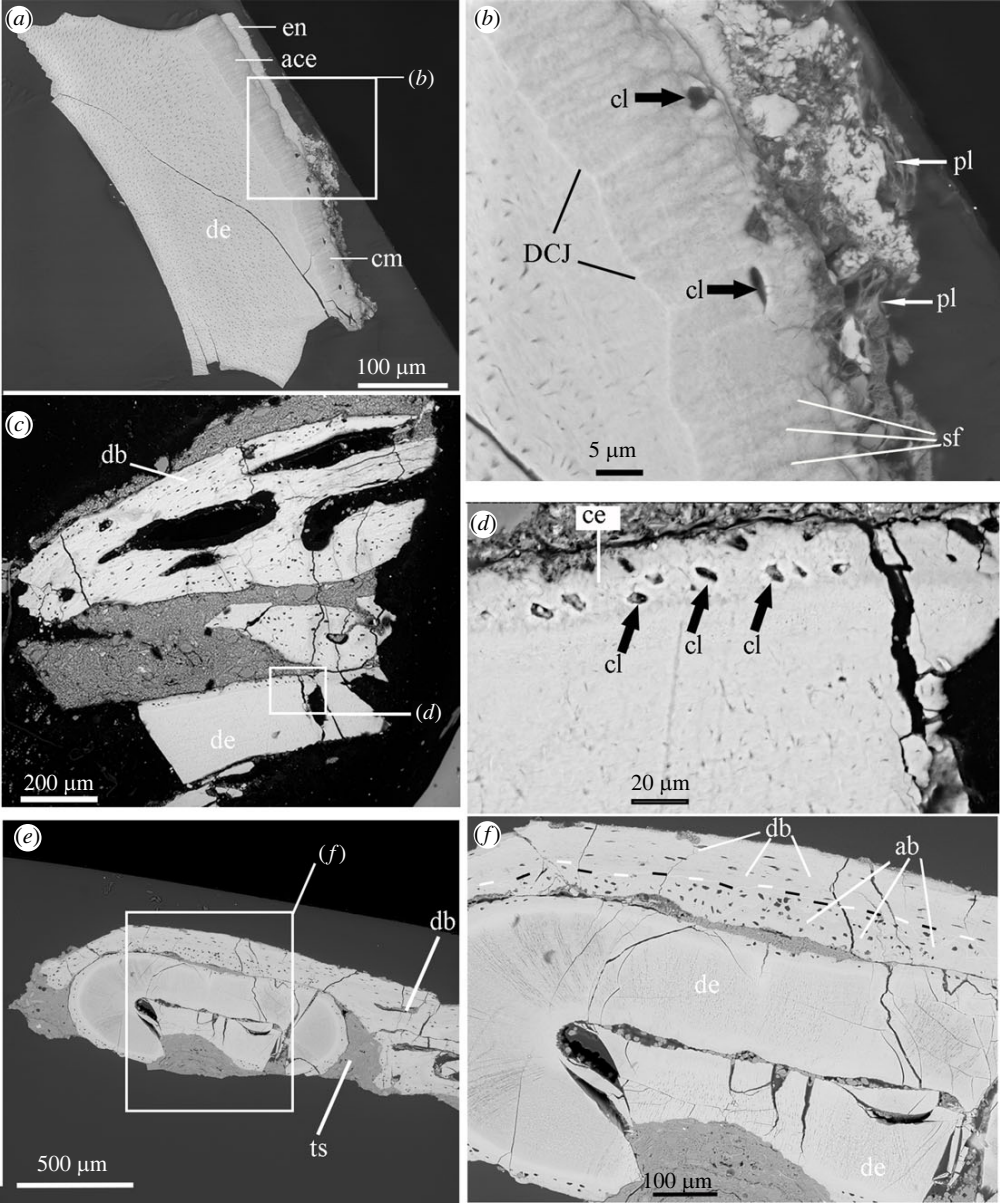
SEM images showing the cementum in the teeth of *Longipteryx* (*a*,*b*) and *Anchiornis* (*c–f*). Note the well-developed cementocyte lacunae (cl) and the brighter region interpreted as the cementing line marking the dentin and cementum junction (DCJ) in *Longipteryx*. Alveolar bone versus dentary bone was demarcated in the cross-section of jawbone with tooth articulated in *Anchiornis* (*e*,*f*) labelled by black-white dash line. Abbreviations: ab, alveolar bone; ace, acellular cementum; cl, cementocytes lacunae; db, dentary bone; de, dentin; DCJ, Dentin-cementum junction; en, enamel; pl, periodontal ligament; sf, Sharpey's fibres; ts, tooth socket.

**Figure 7.**
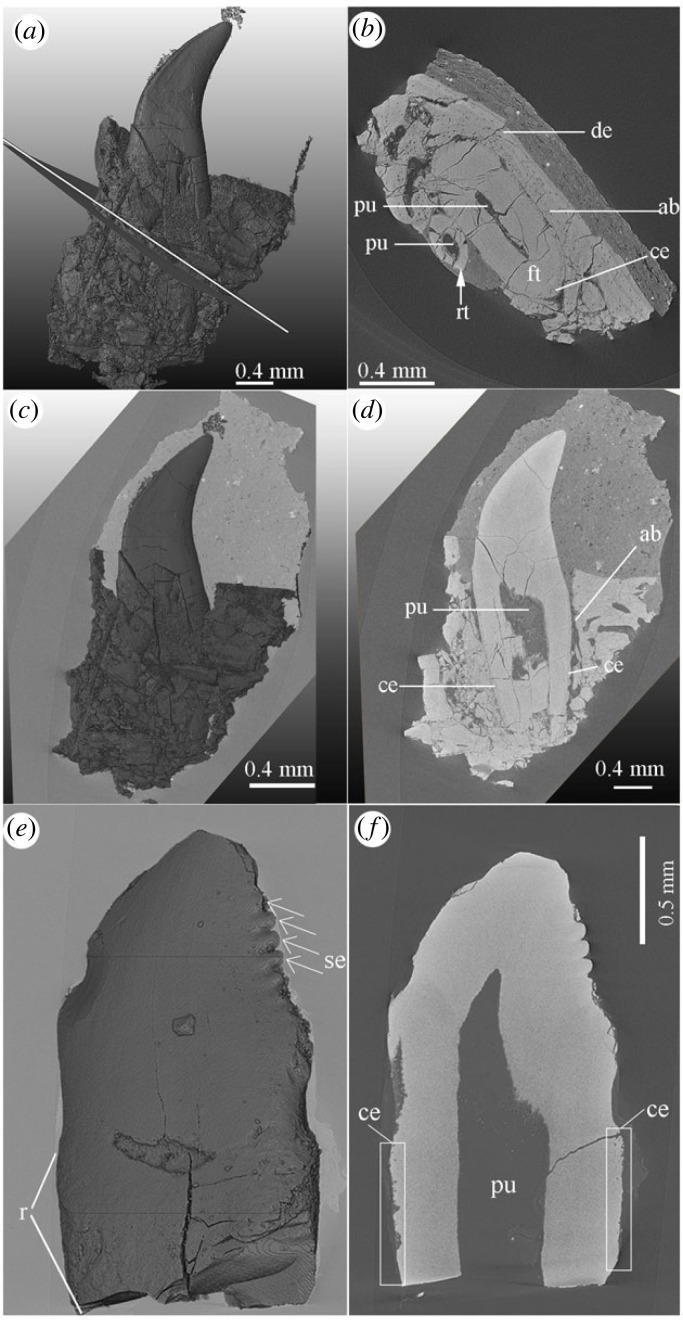
CT rendering with cross and sagittal slices of the tooth sampled from the referred specimen of *Anchiornis* (STM 0-69) (*a*–*d*), and *Microraptor* (STM 5-53) (*e*,*f*). Abbreviations: ab, alveolar bone; ce, cementum; de, dentary; ft, function tooth; r, root; se, serration from the crown; pu, pulp; rt, replacement tooth.

The microscopic features affecting tooth attachment in the alveoli are revealed through CT scans of rostra with *in situ* teeth and SEM of the related sections sampled from the extracted teeth (figures 6 and 7). Abundant vascular foramina of the dentary bone were revealed in *Anchiornis*, which indicate the presence of well-developed passages for blood vessels in the alveolar bone. In a sectioned partial rostral dentary of *Anchiornis* with the attached tooth in the socket, variable features of the osteocyte lacunae (or irregular foramina) were revealed between alveolar and jawbone. The osteocyte lacunae in the alveolar bone are rather round, different from the flat and oval ones in the jawbone (figure 6*f*). The cementing line within the *Anchiornis* jaw can be easily recognized as electronically dense in SEM (using back-scattering diffraction mode), consistent with a greater amount of resorption and remodelling at this level in the dentary bone tissue (SEM).

In addition, compared with the bird teeth examined here, the individual tooth of *Anchiornis* bears a proportionally longer root with well-developed cellular cementum for the attachment of the alveolar bone (figure 6; electronic supplementary material, figures S2–S4). In one complete, isolated *Anchiornis* tooth, the cementum-covered portion of the tooth (or the root) measured approximately 1.51 mm (with a total height of the whole tooth being around 3.0 mm), which is close to the height of the crown (figure 7). The deep inset of *Anchiornis* teeth as indicated by the root length and the cementum suggests very solid implantation within the dentary socket (figure 7). Compared with *Anchiornis*, the alveolar bone in *Longipteryx* dentary were composed of fewer nutrient foramina. This difference could suggest slower tooth replacement in *Longipteryx* and a reduction of other associated dentinal tissues, e.g. cementum in birds (figure 6*a*,*b*). The cementum observed in the *Longipteryx* tooth is rather limited, only extending along part of the root for a distance equal to approximately less than half the length of the tooth crown (electronic supplementary material, figure S4), and the root length is approximately one third of the whole tooth height (root, 0.5 mm; whole tooth height, 1.7 mm). The tooth root is inserted shallowly in the narrow plate-like dentary bone and thin premaxillae through a partially preserved periodontal ligament and visible Sharpey's fibrils (figure 6: pl and sf). The ligamental attachment of tooth in alveolar bone of *Longipteryx* is shared with non-avian dinosaurs and *Hesperornis* reported previously [7]. The proportionally shorter root (compared with the crown, less than half) of the *Longipteryx* tooth for the insertion in the dentary is consistent with the limited development of the cementum.

### 3.4. Slow rate of tooth replacement

The relatively slow skeletal growth rate observed in enantiornithines like *Longipteryx* may relate to the reduced rate of tooth growth and replacement observed in some members of this clade [8,33]. The noticeable size difference between the functional tooth in comparison with that of the nearby replacement tooth further supports the slow tooth replacement rate proposed here for *Longipteryx* (electronic supplementary material, figure S3). Based on this, it is inferred that each tooth remained functional for a longer duration than in non-avian dinosaurs. This may be related to the higher rate of tooth replacement in non-avialan dinosaurs [34]. The replacement tooth erupted on the lingual side of the dentary in *Anchiornis*. A rough calculation of the average duration for the formation of a replacement tooth in *Anchiornis* was calculated to be around 37.5–150 days (based on the dentinal thickness of the functional tooth—the erupting tooth / daily growth thickness 1–4 µm [35]). By contrast, the combination of bone growth rate (electronic supplementary material, figure S5) and slow tooth replacement of *Longipteryx* supports that those teeth maintained a long functional duration as the potential cause of the (hypothesized) dentinal sclerosis observed within the sampled tooth. The age of *Longipteryx* upon death is estimated to be less than 1 year, based on the histological examination of the leg bone (electronic supplementary material, figure S5). Therefore, the occurrences of sclerotic dentin at the mantle dentin region of *Longipteryx* suggest reactive dentinogenesis rather than ageing, which may have resulted from stress during feeding. Since the lifespan of *Longipteryx* appears to be much shorter than a human, it is likely that a different process or more of a physiological process with a higher rate of mineralization led to the tubular sclerosis in *Longipteryx*.

## 4. Discussion

### 4.1. Enamel arrangement

The reduced enamel thickness in troodontid teeth and those of other non-avialan maniraptoran dinosaurs (e.g. dromaeosaurids) may relate to the miniaturization of their body size and a general trend of crown-height reduction within the clade [36,37]. Enamel of *Anchiornis* is much thinner than that of other theropods reported before, such as therizinosaurians [15,26], and it is also thinner than the *Microraptor* tooth compared here. The absence of clearly defined incremental lines of growth within the enamel can distinguish the teeth of *Microraptor* from those belonging to *Anchiornis*. Since both *Microraptor* and *Anchiornis* are carnivores, this difference may suggest different feeding ecologies conveyed by unique tooth-function morphology. We suggest *Anchiornis* was an omnivorous predator with smaller bite forces and mainly feeding on softer prey than *Microraptor* [38]. The differences in enamel microstructures suggest that the morphology in each taxon is largely influenced by the phylogenetic and functional constraint in dinosaur evolution [15,39].

### 4.2. Dentin tubular mineralization

The highly mineralized peritubular dentin observed in the tooth of *Sapeornis* has not been previously recognized in a Cretaceous bird. We attribute these different structures, which are the products of dentinogenesis, as evidence of tertiary dentin. These structures are different from primary dentin structures formed by odontoblast processes observed in the dinosaur samples (contra figures 3 and 4). Among toothed birds, the overall shape of the teeth in these two taxa is distinct compared with the ‘peg-like’ conical tooth morphology observed in most taxa, with the robust and spade-like tooth in *Sapeornis* with medial grooves and basal tubercles and the highly recurved dagger-like tooth in *Longipteryx* with crenulated distal margins. Here we demonstrate for the first time that the unique features of these teeth extend to their microstructure in the form of the highly mineralized peritubular dentin in *Sapeornis* and dentinal sclerosis in *Longipteryx* (figure 5), which both occurred at mantle dentin beneath the enamel–dentin boundary. The new data suggest highly variable mineralized processes were at work shaping the dentition of basal birds during the Cretaceous.

The presence of different types of mineralization in the dentin tubular space of *Longipteryx* is recognized as dentinal sclerosis with the presence of centrally located vacuoles [32]. These cytoplasmic extensions of dentinogenesis are well preserved within the crown dentin in *Longipteryx*, representing the first evidence of active dentinal features in a Cretaceous enantiornithine. Fully occluded dentinal tubules occupied by HAP crystals are present in the mantle dentin while approaching the direction of the enamel–dentin boundary (figure 5). Similar structures have been previously observed in human dentin, mostly occurring in the root and inferred to be triggered by some other external stimuli or ageing [40]. The remarkably preserved development of mineralized tissue associated with tubules of dentin along odontoblast processes documented in *Longipteryx* may be induced as a defensive mechanism to prevent external damage from being transmitted into the internal dentin by mineral infilling in the exterior dentin. Hyper-mineralized peritubular structures are observed in a wide range of mammals, thus not providing clear association between this morphology and a specific diet or feeding behaviour [41]. However, it is consistently observed to be more demarcated in older animals. The hyper-mineralization reported here in *Sapeornis* may be an adaptation for granivory (or fruit-feeding) or simply be due to the older age of the specimen from which the tooth was sampled. The recovered areas for dentinal tubular mineralization in *Longipteryx* and *Sapeornis* are both located not far below the EDJ in the middle part of their teeth. The loss of the interglobular space layer within the mantle dentin region of these early avian species may be a triggering factor [9].

Dental reduction evolved numerous times during the Mesozoic evolution of birds, attesting to the reduction in tooth count and distribution of teeth in both the upper and lower jaws [1,14,15]. These new histological sections of teeth provide microscopic evidence that bird teeth were also modified at the microstructural level, with the teeth becoming more shallowly implanted in the dentary, as evidenced from the reduction of the cementum tissue relative to the sampled theropods and the proportionate length of the root and shallow jaw. Regular mineralization of the tubular tissues (mineralized cast) in the dinosaur tooth was revealed here and in previous studies [42], as well as more frequent replacement of teeth in dinosaurs. The different mechanisms for mineralization of the dentin tubular tissues in bird teeth suggest plasticity in tooth development that is probably related to differences in function. This provides additional support for the growing evidence that bird teeth were highly functional in many lineages [12–14]. The features recovered here, which presumably would have reinforced the teeth, raise the possibility that the teeth in both *Sapeornis* and *Longipteryx* would have experienced extra stress at the exterior dentin under the EDJ boundary, further supporting specialized feeding ecology for these toothed granivore and piscivore, respectively. The hyper-mineralization of dentin tubules and the physiological sclerosis of dentin both occurred in the mantle dentin region. This may make the bird tooth stronger by changed hardness and elasticities and a side effect of loss of interglobular dentin in these taxa [43].

## 5. Conclusion

Here we document important microscopic changes in the morphology of the tooth enamel, dentin and cementum between Mesozoic birds and their close dinosaurian relatives. The hyper-mineralization of peritubular dentin tissues in *Sapeornis* is similar to that frequently reported in many extant mammals, which may be an adaptation for feeding on hard plant materials such as seeds, documented in the crop of several specimens [18]. New evidence indicates that *Longipteryx* had a slow rate of skeletal growth and tooth growth as other enantiornithines, which indicates each individual tooth may sustain functional duration longer than that in non-avialan dinosaurs. An unusual region of greater mineralization within the dentinal tubular structures is observed in the crown dentin (close to the mantle dentin region) of *Longipteryx*, which may potentially increase the dentin hardness [44]. These findings support interpretations that bird teeth played an important role in catching and maybe also processing food items in several lineages.

The varying reactive dentin microscopic features observed here reveal previously unrecognized plasticity in the developmental mechanisms controlling tooth microstructure, suggesting that these morphologies are subject to modification in response to external stimulus and possibly ontogeny. The presented data herein so far strongly suggest additional evolutionary adaptations occurred within the dentin that remain to be explored in non-avialan dinosaurs and Mesozoic avialans. Therefore, more work on additional taxa is likely to yield further insight into tooth growth, replacement and function in birds and dinosaurs.

## Data Availability

The CT data of individual tooth (compressed JPEG) are deposited in the open science framework (https://osf.io/3nkcy/). The data are provided in the electronic supplementary material [45].
